# Epigenetics of Thymic Epithelial Tumors

**DOI:** 10.3390/cancers15020360

**Published:** 2023-01-05

**Authors:** Vanessa Nicolì, Fabio Coppedè

**Affiliations:** 1 Department of Translational Research and of New Surgical and Medical Technologies, University of Pisa, 56126 Pisa, Italy; 2 Interdepartmental Research Center of Biology and Pathology of Aging, University of Pisa, 56126 Pisa, Italy

**Keywords:** thymic epithelial tumors, thymoma, thymic carcinoma, DNA methylation, histone tail modifications, ncRNAs, microRNAs, thymoma-associated myasthenia gravis (TAMG), epigenetics, biomarker

## Abstract

**Simple Summary:**

Thymic epithelial tumors (TETs) are rare malignancies that arise from the epithelial cells of the thymus. In most cases, TETs are characterized by a good prognosis, and their aggressiveness reflects the histopathological subtypes defined in the guidelines of the World Health Organization. Over the years, the challenge has been to characterize TETs at both genetic and epigenetic levels, revealing a deep deregulation of key cellular pathways linked to cancer onset and development or autoimmunity. The present work collects the main research concerning the epigenetic deregulation of TETs, with a special focus on DNA methylation, histone tail modifications, and non-coding RNAs dysregulation potentially useful for clinical practice.

**Abstract:**

Thymic epithelial tumors (TETs) arise from the epithelial cells of the thymus and consist in the 1% of all adult malignancies, despite the fact that they are the most common lesions of the anterior mediastinum. TETs can be divided mainly into thymomas, thymic carcinomas, and the rarest ad aggressive neuroendocrine forms. Despite the surgical resection is quite resolving, the diagnosis of TETs is complicated by the absence of symptoms and the clinical presentation aggravated by several paraneoplastic disorders, including myasthenia gravis. Thus, the heterogeneity of TETs prompts the search for molecular biomarkers that could be helpful for tumor characterization and clinical outcomes prediction. With these aims, several researchers investigated the epigenetic profiles of TETs. In this manuscript, we narratively review the works investigating the deregulation of epigenetic mechanisms in TETs, highlighting the need for further studies combining genetic, epigenetic, and expression data to better characterize the different molecular subtypes and identify, for each of them, the most relevant epigenetic biomarkers of clinical utility.

## 1. Introduction

The thymus is the primary lymphoid organ designated to the adaptative humoral immunity construction. It guarantees immune self-tolerance by operating the positive and negative selection of the T cells during their development and maturation, avoiding autoimmune manifestations. The thymus explicates its functions principally during in utero life until puberty and then undergoes a gradual physiological involution while minimal activity is still maintained [1].

Thymic epithelial tumors (TETs) are malignancies arising from the epithelial cell network of the thymus. Although rare (1% of all adult malignancies), TETs are the most common lesions that develop in the upper anterior part of the mediastinum, seldom they were described ectopically located in middle and posterior spaces, neck, lung, and pleura [2]. TETs can be distinguished in thymomas (TMs), thymic carcinomas (TCs), and the very uncommon neuroendocrine carcinoids of the thymus (NETs) [3], which differ each other for tumorigenic potential.

Epigenetic modifications, including changes in DNA methylation, post-translational modifications on histone tails, and a dysregulated expression of non-coding RNA molecules (ncRNAs), result in altered gene expression levels and are increasingly regarded as contributors to tumorigenesis as they can lead to genome instability and chromosome aberrations, activation of transposable elements, over-expression of proto-oncogenes, and silencing of tumor suppressor genes [4,5,6]. Indeed, there is an increasing interest in the identification of cancer epigenetic biomarkers and their potential application in clinical settings as either diagnostic or prognostic biomarkers or as novel targets for therapeutic interventions [7,8,9,10,11].

In this review, we summarize the knowledge about epigenetic modifications occurring in TETs and discuss their potential utility as clinical biomarkers.

## 2. Thymic Epithelial Tumors

According to the RARECARE project data [12], TETs present an overall annual incidence rate of 0.18/100,000 in the European population, with a debated slight preponderance (1.4:1) in males [13]. A similar incidence of about 0.13–0.15 per 100,000 person-years has been reported in the United States [14,15], with higher rates among Asian Pacific Islanders and Afro-Americans than white people [16]. A higher incidence of TETs has been reported in Asian populations, accounting for 0.39–0.50 per 100,000 person-years [17,18]. Moreover, differences concerning disease age at onset are observed among different ethnic groups. For example, the onset of TETs is frequently linked to aging in Caucasians, with both TMs and TCs peaking around the age of 60–70 years [16]. On the other hand, a median age of 48 years at the TMs onset was reported among Afro-Americans, also suggesting the rooted involvement of genetic factors in the pathogenesis of the disease [15]. However, accurate estimations of TETs incidence are complicated by the common absence of symptoms in the affected population. In fact, TETs result in fully asymptomatic in 30% of the cases, and an accidental diagnosis is not uncommon during a chest radiography or computed tomography (CT) scan. In the symptomatic cases, the spectrum of symptoms includes different manifestations, with patients experiencing respiratory complications due to the intrathoracic compression brought on by the mass or, rarely, systemic symptoms [13].

Morphologically, TETs are characterized by a great heterogeneity. For instance, low-risk TMs are characterized by round shapes with smooth borders, in contrast to high-risk TMs and TCs that show irregular shapes and borders. In these cases, fat invasion beyond the borders of the lesion, mediastinal vascular and pericardial invasion to neighboring lung tissue, mediastinal lymph node enlargement, and thoracic cavity and pericardial infiltration have been reported [19,20]. Thus, establishing a standard for clinical or histopathological classifications has been notoriously difficult, and several revisits of the guidelines were made over the years [20]. Criteria used especially consider the rate of non-neoplastic thymic epithelial cells and lymphocytes components, the tumor behavior, the clinical landscape, and the prognosis of tumors, which are useful features to drive postoperative treatments [21].

The Masaoka system, integrated with Koga’s modifications and revisited in the following years, represents the most widely adopted staging system for TETs. However, the tumor, node, metastasis (TNM)-based classification has been proposed by the International Association for the Study of Lung Cancer (IASLC)/International Thymic Malignancy Interest Group (ITMIG) in 2014 to conform TETs staging with other types of cancer [22,23].

Based on the World Health Organization (WHO) classification of tumors of the thymus, TMs are divided according to their histology into type A (relative mean incidence 11.5%), AB (relative mean incidence 27.5%), and B1, B2, and B3 subgroups (relative mean incidence 17.5%, 26%, and 16% respectively) [3,19]; but it is not uncommon to find TMs composed of more than one histologic type. Moreover, rare forms of TMs, such as micronodular thymoma, sclerosing thymoma, and metaplastic thymoma, were documented [24,25,26,27]. The severity of TMs reflects the histological type, albeit they are slow-growth tumors with a good prognosis.

Indeed, types A, AB, and B1 have a very good prognosis, with reported overall survival (OS) rates ranging from 90% to 95% at ten years, whilst B2 and B3 thymomas show OS rates of 75% and 70% at five years, respectively [28]. The OS increases in resectable thymomas (5/10-year disease-free survival rates were approximately 80%), and the recurrence rate after the complete resection ranges from 5 to 50% depending on TM stages [29,30].

In contrast, TCs and NETs are characterized by more aggressive behaviors, less responsiveness to chemotherapy, and a tendency for a metastatic spread in the liver, nodes, bone, lung, or brain [20,21,22]. In particular, TCs represent 10–20% of all TETs [19] and show a 5-year OS of 48% [28]. Most of them arise de novo, but some arise through a transformation from a TM. A large variety of TCs is described in the literature, divided into low- and high-grade carcinomas [3,31]. Similarly, NETs account for 0.4% of all carcinoid tumors and can be distinguished into well-differentiated NETs (low-grade; atypical and typical carcinoid) and low-differentiated ones (high-grade; small-cell and large-cell NETs) [32]. In total, 50% of NETs manifestations are associated with endocrinopathies, including Cushing’s syndrome, acromegaly, or multiple endocrine neoplasia-1 (MEN1), in which NETs represent the major cause of death [32,33].

Interestingly, neural autoantibody positivity often accompanies TETs, with or without manifesting associated autoimmune paraneoplastic disorders (ADs) [34,35,36,37]. Among them, myasthenia gravis (MG) is observed in 30% of TMs (TAMG = thymoma-associated myasthenia gravis), while lupus erythematosus (LE), pure red cell aplasia (PRCA), and other diseases characterized by an autoimmune component are less frequently observed (1–2%). TMs in the advanced stage (B1 or B2) are more prone to develop ADs, which are fairly different from those observed in TCs or NETs [38].

The pathophysiological link between TETs and autoimmunity lies in the nature of the tumor itself. TETs are functional lesions capable of producing immature CD4+/CD8+ T double-positive cells, as it happens in the cortex of the normal thymus [39]. One of the theories proposed states that the negative selection (that occurs in the medulla after the positive selection) fails or results insufficient in the TETs. The reasons are attributed to the deficiency of the autoimmune regulator (*AIRE*) and the forebrain-expressed zinc finger 2 (*FEZF2*) gene products, and to the absence of bone-marrow-derived dendritic cells [39,40]. Moreover, also the impairment of the human leucocyte antigen (*HLA-DR*) expression in neoplastic epithelial cells of TMs possibly affects the positive selection of CD4+/CD8− single-positive T cells [41]. Thus, defects in the selection steps result in both the maturation of autoreactive cells and their delocalization in the peripheral sites [42].

The scenario ahead, when talking about TETs, is evidently convoluted, and the genetic landscape, resulting from the investigation of TETs, reflects their complexity. For instance, general transcription factor IIi (*GTF2I*) mutations are the most frequent in TMs (39%) but not in TCs. These, together with mutations of *HRAS*, *NRAS*, and tumor protein P53 (*TP53*), genes are defined as “founder mutations” since they occur in the very early stages of malignancy [43]. Instead, TCs have a different genetic profile, showing recurrent somatic mutations also in ten-eleven translocase 2 (*TET2*), CYLD lysine 63 deubiquitinase (*CYLD*), SET domain containing 2 (*SETD2*), F-box and WD repeat domain containing 7 (FBXW7), and retinoblastoma transcriptional corepressor 1 (*RB1*) genes [44]. Mutations in the epigenetic machinery genes have been also found in TETs. Less is known concerning the molecular profile of NETs, which was investigated in a limited number of studies recently summarized [45,46].

TETs diagnosis and staging are based on imaging approaches. Chest radiographs can be the first modality suggesting a thymic mass, and computed tomography (CT) is the preferred choice for imaging thymic tumors, as it can discern the location, morphology, size, shape, margins, and invasion of adjacent structures. Magnetic resonance imaging (MRI) is not routinely used for the evaluation of TETs but can allow us to distinguish solid from cystic lesions in the case of equivocal CT scans. Positron emission tomography (PET)/CT combines the anatomical details of CT with the metabolic information of PET and can allow differentiating aggressive tumors, such as TCs, due to their higher metabolism or detection of metastases [47,48]. Concerning the prognostic factors for TETs, there is a consensus that advanced histological WHO types, as well as advanced Masaoka-Koga or TNM stages, are all associated with a worse prognosis. Many additional prognostic factors have been proposed for TETs, including age, tumor localization, presence of MG, recurrence-free period, number of recurrent lesions, etc. However, the high heterogeneity of TETs has sometimes led to discrepancies among studies [49]. Surgical, radiation, and systemic treatments are available for patients with TETs. Surgical resection is the mainstem treatment for most TETs, eventually followed by further post-operative (adjuvant) treatments. Indeed, complete excision of the tumor represents the best prognostic factor in TMs [49]. Platinum-based chemotherapy is considered the standard of care for advanced (non-resectable) or metastatic disease [49,50,51]. Immunotherapy increases the risk of immune-related adverse events, especially in patients with TMs, and therefore can be considered only in some cases of TCs [52].

TETs therapeutic options have been recently and systematically reviewed [53]. More than 100 studies were included in that systematic review, and their meta-analysis revealed an overall survival benefit with postoperative radiotherapy for patients with TMs or TCs. Less clear was the effect of neoadjuvant chemotherapy on overall survival, and the authors suggested that this could be due to the literature’s bias [53]. The results of that systematic review have led to the production of clinical practice guidelines with detailed, evidence-based recommendations to improve patient’s management [54]. An integrated molecular characterization of the genetic and epigenetic landscape of TETs could represent a promising approach for the identification of novel prognostic biomarkers and for the development of targeted therapies [49].

## 3. Epigenetic Mechanisms

**Figure 1:**
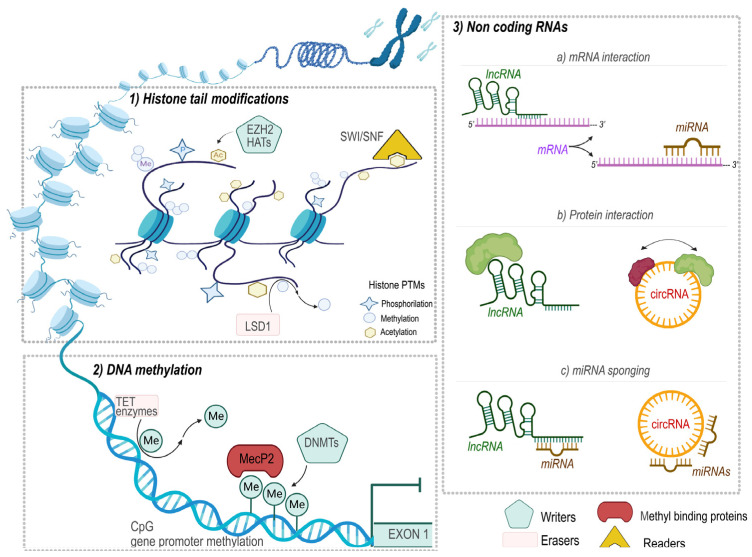
The epigenetic mechanisms and the key players (writers, erasers, readers, and methyl-binding proteins) of the epigenetic regulation are shown in Figure 1. (**1**) Post-translational modifications (PTMs) occur at the level of histone tails and influence the chromatin accessibility to various transcription factors. Among the modifications that could occur, acetylation and methylation are some of the best characterized. Moreover, the figure shows examples of the writer enzymes enhancer of zeste homolog 2 methyltransferase (EZH2) and histone acetyltransferases (HATs), which promote, respectively, methylation and acetylation on histone tails, which are read by specific enzymes, such as SWI/SNF, an H3K27 acetylation reader. Finally, lysine specific demethylase 1 (LSD1), among several others, is the enzyme responsible for the removal of histone modifications, and particularly some methylation marks. (**2**) DNA methylation occurs at level of the C5 position of the cytosine. Methyl groups are added by specific DNA methyltransferases family enzymes (DNMTs), erased by the ten-eleven translocation methylcytosine dioxygenases (TET enzymes), and read by methyl CpG-binding proteins, such as MeCP2. (**3**) Non-coding RNAs can be divided in various classes based on their length and structure. The figure shows microRNAs (miRNAs), long non-coding RNAs (lncRNAs), and circular RNAs (circRNAs). NcRNAs orchestrate different mechanisms, including: (**a**) post-transcriptional gene regulation through the interaction with the untranslated region of the mRNAs and the sequestering of them; (**b**) both lncRNAs and circRNAs could interact with the cytoplasmatic proteins influencing their half-life or promoting the interaction between proteins; (**c**) ncRNAs could bind other ncRNA species (miRNAs sponging) inhibiting/promoting their functions.

The epigenetic regulation of gene expression is a hot topic strongly linked to cancer development [55,56,57,58]. The ability to alter gene function, in the absence of mutations in the DNA sequence, is the main feature of the epigenetic machinery, which confers to them a deep dynamic nature. This plasticity allows these mechanisms to involve a wide range of both physiological and pathological processes, including cancer development and progression. In this context, the key players in epigenetic regulation are DNA methylation, histone post-translational modifications (PTMs), and mechanisms mediated by ncRNAs (Figure 1). The epigenetic pathways are orchestrated by the action of a plethora of enzymes able to “write”, “remodel”, and “erase” the epigenetic marks on nucleic acids or proteins [59,60,61].

### 3.1. DNA Methylation

DNA methylation is the best characterized among the epigenetic mechanisms. It consists of an alkylation reaction, where a methyl group (CH3) substitutes a hydrogen atom on the fifth carbon of cytosines, leading to the formation of 5-methylcytosine (5 mC) on symmetrical 5′-CpG-3′ dinucleotides, denoted as cytosine-phosphate-guanine (CpG) sites. The transfer of the methyl group from the donor S-adenosylmethionine (SAM) to cytosine is mediated by the DNA methyltransferase (DNMT) family of enzymes, of which, each member has a peculiar function [62]. For instance, DNMT1 is located near the replication fork and is denoted as the maintenance DNMT, as it guarantees the maintenance of the methylation patterns during DNA replication, while DNMT3A and DNMT3B are involved in the de novo establishment of methylation signatures during development and tumorigenesis. More recently, the de novo methyltransferase enzyme DNMT3C, which is highly specialized in the methylation of young retrotransposon promoters with essential involvement in male fertility, has been identified in murine germ cells [63,64]. Unlike the other DNMTs, DNMT2 is an RNA methyltransferase specific for transfer RNAs (tRNAs) [65,66]. The ten-eleven translocation enzymes (TET enzymes) are the proteins involved in the active DNA demethylation processes, mediating the successive oxidation of 5 mC to 5-hydroxymethylcytosine (5 hmC), 5-formylcytosine (5 fC), and 5-carboxylcytosine (5 caC) [67].

CpG islands are regions extremely rich in CpG sites located within the promoter region of almost 70% of human genes, and their methylation affords a specific binding site for a wide range of proteins (methyl-CpG-binding proteins) that trigger methylation-mediated transcriptional repression. Moreover, DNA methylation of the promoter region can directly impair gene transcription, making it difficult the access of the transcriptional machinery [68,69,70,71].

Thus, DNA methylation is a reversible and dynamic process [72]; its impairment leads to a global or gene-specific hypomethylation or hypermethylation and the loss of imprinting, alterations that are frequently detected in cancer onset and progression [73,74,75,76,77,78].

### 3.2. Histone Post-Translational Modifications

In eukaryotes, genomic DNA curls around a group of proteins called histones, core units that constitute nucleosomes, forming the following dynamic nucleoprotein structure: chromatin. Basically, a nucleosome consists of 147 base pairs of DNA wrapped around an octamer composed of two copies of each histone protein (H3, H4, H2A, and H2B) [79]. A nucleosome is structured by a central H3-H4 tetramer flanked by two H2A-H2B histone dimers [79]. Moreover, the nucleosomes are connected by a short strand of DNA stabilized by the H1 linker histone proteins [80].

Histone proteins are susceptible to several PTMs that occur mainly, but non-exclusively, along with their bulged unstructured N-terminal tails. Methylation, acetylation, phosphorylation, and ubiquitination are the best characterized among the distinct modifications that occur [81]. However, the chromatin state is governed by the cooperation of all the histone PTMs, including sumoylation, ADP-ribosylation, carbonylation, deamination, formylation, O-GlcNAcylation, propionylation, butyrylation, crotonylation, and proline isomerization [82].

The presence of specific histone PTMs, together with DNA methylation, is indicative of the transcriptional state of the genome. In particular, histone hyperacetylation and the di-tri-methylation of the histone 3 lysine 4 (H3K4) and H3K36 are the peculiar epigenetics tags of the euchromatin characterized by a relaxed chromatin state and an active transcription [83]. In addition, the specific position of histone PTMs and the degree of modification affect the resulting control. For instance, H3K4 methylation, operated by the Complex of Proteins Associated with Set1 (COMPASS), is found in its monomethylated form (H3K4me1) at the enhancers; it is demethylated (H3K4me2) among the 5′ of the transcribing genes, while the trimethylation (H3K4me3) is a peculiar of the promoter of the active genes [59].

Moreover, distinct marks allow for discrimination between constitutive versus facultative heterochromatin. The peculiar modification of the constitutive heterochromatin is the trimethylation of the H3 histone (H3K9me3), operated by the Suv39h family enzymes, and the absence of histone acetylation [84]. Constitutive heterochromatin controls telomeric and pericentromeric chromosomal regions, as well as transposable elements and other virus-derived sequences. On the other hand, the H3K27me3 and H2AK119 monoubiquitination are the repressive marks that usually enrich facultative heterochromatin. These modifications are operated by the Polycomb (PcG) proteins, which are further subdivided into the following two different complexes: PRC2 contains the H3K27 methyltransferase EZH2 (and many other proteins), and PRC1 is an E3 ubiquitin ligase with specificity for H2AK119 [85]. Interestingly, PCR2 and PCR1 cooperate, establishing a positive loop by recruiting several readers proteins that trigger the activity of the complexes [85]. Facultative heterochromatin is scattered on the genome and controls several physiological processes, such as X chromosome inactivation, imprinting at specific loci, and promoting homeotic gene cluster repression [83]. Despite heterochromatin being commonly associated with transcriptional silencing, it was recently proposed its contribution to the activation of some genes and in the expression of non-coding RNA transcripts and of the proteins binding these RNAs [85].

Thus, chromatin is not an inert structure, but a sensitive structure defining the accessibility of DNA in response to various stimuli through the key role of histone PTMs and their orchestrated interaction with other epigenetic mechanisms.

### 3.3. Non-Protein Coding RNAs

Non-coding RNAs (ncRNAs) are short fragments of RNA molecules that are transcribed but not translated into proteins and therefore considered at first as “junk” or experimental artifacts.

NcRNAs were almost recently included in the epigenetic regulation mechanisms for their capability to affect protein expression without DNA sequence changes [86].

Considering their length, ncRNAs can be classified as long non-coding RNAs (lncRNAs), which are composed of about 200 nucleotides, reorganized in different configurations; the two main groups are linear and circular lncRNA, and are processed in nuclear or cytosolic compartments via the same machinery of mRNA transcription [87]. Linear lncRNAs are implicated in a wide range of mechanisms, including the maintenance of nuclear structure integrity, the transcription factors, or chromatin-modifying complex recruitment, interaction with RNA-binding proteins, or acting as competing endogenous RNAs (ceRNAs) for miRNAs [87]. Circular lncRNAs (circRNAs) regulate the expression of target genes, acting as a sponge for miRNAs and transcriptional factors [88]. On the other hand, short non-coding RNAs (sncRNAs) are characterized by 18–25 nucleotides in length and toughly classified based on their genomic origins and mechanisms of action. Among the sncRNAs, miRNAs are the best-studied subgroups, which participate in gene regulation at transcriptional levels, as well as at the post-transcriptional one [89]. The biogenesis of these short RNA molecules starts in the nucleus, where they are transcribed by DNA polymerase II and further processed in a pre-miRNA by the specific ribonuclease Drosha complex. Its maturation proceeds in the cytosol, and the mature molecules could be incorporated into the RISC (RNA-induced silencing complex) or follow the Argonaute (AGO) catalysis to reach the mRNA target [90]. Different isoforms of the AGO family exist in humans, all of which are competent for gene silencing. In particular, AGO2 loads the small interfering RNA (siRNA) and miRNA into the RISC complex to achieve its RNA interference activities [91].

Usually, miRNAs recognize a complementary 2–7 nucleotides long sequence of the target that can be located in 3′UTR, 5′UTR, or in the coding region of the mRNA [92]. The resulting regulation depends on exactly the location of the recognition site as follows: a down-regulation or the complete suppression of the protein encoded by the messenger molecule will happen in the case of 3′UTR recognition. On the contrary, a specific recognition site on 5′UTR or in the coding region of mRNA triggers the upregulation of the protein. Interestingly, their expression is epigenetically regulated by methylation within the promoter region or along the RNA sequence itself (i.e., N6-methyladenosine (m6A), 5-methylcytosine (m5C), deamination) and histone modifications [92].

Thus, ncRNAs are epigenetic regulators that can respond to the cellular environment and produce the correct responses to the stimuli. These molecules are involved in different regulatory cellular pathways, including cell cycle, apoptosis, chromatin changes, differentiation, and many others [93].

## 4. DNA Methylation in Thymomas and Thymic Carcinomas

In the past years, several studies have explored the tumor genome in search of methylation markers with potential clinical significance. Data concerning TMs and TCs are summarized in Table 1.

### 4.1. Thymomas

Recently, the genome-wide methylation study conducted by Bi’s team [103] allowed the identification of 119 hypermethylated and 18,999 hypomethylated CpGs in 6 biopsies of TMs paired with normal adjacent tissues, revealing a general hypomethylation in tumor tissues. Moreover, the study proposed that the TMs types A vs. B macro groups could be epigenetically distinguishable by a total of 10,014 differentially methylated CpGs (DMCs). The majority of these CpGs were located in the gene body and belonged to the open sea area of the genome. Furthermore, methylation data have been combined with expression array data from the GEO database identifying seven genes that were hypermethylated with low expression (e.g., intercellular adhesion molecule 3 (*ICAM3*), amyloid beta precursor protein-binding family B member 1 interacting protein (*APBB1IP*), interferon gamma-inducible protein 16 (*IFI16*), parvin gamma (*PARVG*)), and 29 hypomethylated genes with high expression (e.g., zinc finger protein 396 (*ZNF396*), transmembrane protein 237 (*TMEM237*), fraser extracellular matrix complex subunit 1 (*FRAS1*), ribosomal protein L22 (*RPl22*), fasciculation and elongation protein zeta 2 (*FEZ2*), epidermal growth factor receptor pathway substrate 15 (*EPS15*)). Eleven of these genes allowed discrimination of A vs. B subtypes with an overall accuracy of >80% [103]. The cellular pathways acknowledged the affect of methylation in TETs, including focal adhesion, regulation of actin cytoskeleton, natural killer cell-mediated cytotoxicity, calcium signaling, and other cancer-related pathways [103]. Two recent studies used DNA methylation data available in the Cancer Genome Atlas (TCGA) database to compare DNA methylation differences between TMs and TCs, revealing several epigenetic differences that will be detailed in the following section of this article [102,111]. Several previous studies based on the candidate gene approach have shown that tumor suppressor genes, such as human mutL homolog 1 (*hMLH1*), O-6-methylguanine-DNA methyltransferase (*MGMT*), cyclin-dependent kinase inhibitor 2A (*CDKN2A*), ras association domain family member 1 (*RASSF1A*), and many other genes are less frequently methylated in early-stages TMs, as compared to invasive TMs and TCs [94,95,98,99,101]. For example, specific hypomethylation was found in the promoter region of nine genes involved in critical cellular pathways (e.g., regulation of cell cycle progression, DNA repair complex) deregulated during cancer development, including *hMLH1*, *MGMT*, fragile histidine triad diadenosine triphosphatase (*FHIT*), adenomatous polyposis coli promoter 1A (*APC1A*), retinoic acid receptor beta (*RARβ*), and E-cadherin (*E-cad*), among the low-grade TMs (types A/AB/B1). *hMLH1*, *MGMT*, and *E-cad* are genes linked to DNA repair mechanisms, while *FHIT*, *APC1A*, and *RARβ* are tumor suppressor and “caretaker” genes, respectively [113,114,115,116]. Interestingly, cancer progression along the more severe forms (B2/B3/C) was signed by the hypermethylation of the promoters of the genes mentioned above, followed by the transcriptional silencing of some of them [98]. Similarly, the tumor suppressor gene *FBXW7* was more frequently methylated in B-type than in A- or AB-type TMs [96]. More recently, another work investigated the promoter methylation level of *RASSF1A*, *hMLH1*, *MGMT*, and cyclin dependent kinase inhibitor 2A (*CDK2NA*) genes in the blood and tissues of 69 TAMG patients. The analyses were also performed in the adjacent healthy tissues that were available from 44 of them [104]. The results obtained in this work revealed substantial demethylation of the investigated genes in all the tissues considered, without detectable differences among them. Thus, no correlations of potential clinical utility were found with histologic or pathologic features. Furthermore, the methylation status of the promoter region of methylenetetrahydrofolate reductase (*MTHFR*), *DNMT1*, *DNMT3A*, and *DNMT3B* genes was investigated in the same TAMG cohort [100]. MTHFR is a key enzyme in folate metabolisms, and as the DNMTs, it is involved in DNA methylation, as well as in DNA repair and synthesis. The enzymatic activity of this enzyme could be affected by genetic variation and contributes to several cancer types’ development and progression [117,118,119,120].

Among these genes, *MTHFR* and *DNMT3A* promoters showed different methylation levels between blood and tumor tissue. In particular, the *MTHFR* gene resulted more methylated in tumors with respect to the adjacent healthy tissue, with a strong correlation between methylation levels in the blood and tumor biopsy. Moreover, this evidence was further corroborated by a recent investigation based on the TCGA database of TETs. The authors observed a negative correlation between *MTHFR* methylation and expression levels in TETs, and speculated that the lower activity of the *MTHFR* enzyme, also affected by the *MTHFR* C667T gene polymorphism, causes a significant DNA hypomethylation in TETs that promotes the activation of proto-oncogenes [108].

Controversial results were obtained for growth hormone secretagogue receptor (*GHSR*) gene methylation levels in TMs, which is considered a pan-cancer biomarker. The *GHSR* gene encodes for the receptor of the ghrelin hormone, which principally modulates the energy balance, appetite, insulin secretion, and gastric fitness [121]. However, ghrelin and its receptor are shown to be highly expressed in several cancer types as their role in cancer proliferation, inflammation, metastases formation, and prediction of clinical outcome have been investigated [121].

*Kishibuchi* and colleagues (2020) [106] found methylation of the CpG sites located downstream of the transcriptional start site of the *GHSR* gene among all TM and TC tissues analyzed with respect to healthy tissues, with no significant differences noted among TM subtypes. Analyzing the expression levels of five key components of the ghrelin system in 58 TETs, a positive correlation between *GHSR* methylation in TMs and the increase in mRNA expression of *In-1 ghrelin* (splicing variant) and *GHSR1b* (receptor variant) existed with respect to adjacent healthy tissues. These results suggest that *GHSR* methylation promotes the variant forms of naïve ghrelin, contributing to TMs onset and progression [122]. In contrast, another study detected hypermethylation of the *GHSR* promoter in 28% of the 65 analyzed TMs from TAMG patients, particularly in advanced stages [105]. Interestingly, the genome-wide methylation study by Bi and co-workers revealed that TMs from TAMG patients have a distinct methylation profile compared to TMs from individuals without MG, but the study included only six samples, so the results warrant confirmation in larger datasets [103]. Another potential pan-cancer methylation biomarker was identified through the analysis of the TCGA dataset. In particular, authors observed hypermethylation of the MARVEL domain containing 1 (*MARVELD1*) locus that affected its expression levels in various cancers, including TMs [112].

On the other hand, a low-grade TMs biomarker was recently identified in a gene named KIT proto-oncogene ligand (*KITLG*), a pleiotropic factor involved in cell proliferation, differentiation, and survival. Using *KITLG*-related genomics and methylation data for TMs in the TCGA and GEO databases, the authors established that *KITLG* was highly expressed in type A and AB TMs; moreover, a specific hypomethylation in differential methylated regions (DMRs) seems to be peculiar of TMs with *KITLG* overexpression, also accompanied by DNMT3B higher expression [107].

An elegant work conducted by Kont’s team compared the DNA methylation of *AIRE* promoter in normal thymic epithelial cells derived from TMs and normal tissues [123]. *AIRE* codes for a transcriptional regulator that is required for the negative selection of the autoreactive T-cells in the thymus. The analyses showed a particular heterogeneity of the methylation pattern that was independent of the histological type of TMs, with a prevalence of hypomethylation specific to tumor tissues in most of the sites analyzed. However, individual variable patterns weaken the hypothesis that the downregulation of *AIRE* expression occurs by its CpG promoter methylation alone. Moreover, specific methylation signatures were found in the different cell types of the thymus [123,124].

In summary, several DNA methylation markers have been proposed for TMs, and the aberrantly methylated genes are involved in different cellular pathways related to cancer onset and progression. Most of the available literature has focused on the potential use of DNA methylation markers to discriminate among different histologic subtypes, between TMs and TCs, or between TMs from TAMG patients and those from individuals without MG. A prognostic role for certain DNA methylation markers has been also suggested in TMs [90], and this is further detailed in the following section by the description of studies that compare early and advanced TET stages.

### 4.2. Thymic Carcinomas

TCs show unique clinicopathological features and genetic profiles that make them very different from TMs. Similar evidence has emerged from several studies aimed at exploring the methylation levels of TETs. TCs biopsies were also included to validate methylation as a biomarker for the OS in patients with TETs or to find specific traits that can distinguish TMs from TCs (Table 1).

Some investigators compared genome-wide DNA methylation differences between TMs and TCs in order to identify the specific methylation signatures of these disorders. Li and coworkers [102] analyzed DNA methylation data from 124 tumor tissues available in the TCGA database, including 13 type A–B3 and 11 types C TETs. The study found 5155 TC-specific hypomethylated CpG sites (corresponding to the genes involved in focal adhesion and apoptosis) and 6967 hypermethylated CpG sites (comprising the genes principally involved in neuroactive ligand-receptor, calcium signaling, and cAMP signaling pathways). Among them, 187 CpG sites were identified as potential DNA methylation markers for OS. Four genes, kinase suppressor of Ras 1 (*KSR1*), E74-like ETS transcription factor 3 (*ELF3*), interleukin 1 receptor antagonist (*IL1RN*), and recombination activating 1 (*RAG1*), were selected for their sensitivity, but the *ELF3* and *IL1RN* gene promoter methylation showed major sensitivity in predicting OS in the patients. *ELF3* is a gene involved in inflammatory responses and may promote epithelial cell proliferation and tumorigenesis [125], and *IL1RN* is a key inflammatory mediator [126]. In Li et al., 2019 [102], the authors speculate that the strongest link with OS in the patients was probably due to their involvement in the immune regulation of the thymus malignancy [102].

Based on the genome-wide approach, another group identified 92 hypermethylated CpG islands in TCs, comparing 7 TC samples with 8 B3 TMs. In a replication cohort composed of 46 TETs and 20 paired normal thymus tissues, authors confirmed hypermethylation of the promoter of *GHSR*, G protein subunit gamma 4 (*GNG4*), spalt-like transcription factor 3 (*SALL3*), and homeobox D9 (*HOXD9*) genes in TCs compared to TMs. At the same time, only *HOXD9* and *SALL3* were strongly linked with worse relapse-free survival [106]. In another study, the same group found that the median DNA methylation rate of the glutamate decarboxylase 1 (*GAD1*) gene promoter was significantly higher in the TC samples than in the TM ones (73 TMs and 17 TCs). Moreover, the higher *GAD1* methylation, as well as mRNA and protein expression, resulted specifically for TCs compared with both TMs and normal tissues, being indicative of the more aggressive phenotype of TETs [109].

A more recent analysis of the TCGA dataset identified 40 upregulated and 179 silenced genes, as well as more than 500 DMC, between TMs and TCs. Among them, methylation of cg20068620 in mitogen-activated protein kinase 4 (*MAPK4*) was significantly associated with recurrence-free survival (RFS) in both the 124 TETs included in the TCGA dataset and in a replication cohort of 95 TET patients [111].

An integrated analysis of the genetic and epigenetic characterization of 10 TC pairs with healthy tissues revealed that TCs with recurrent somatic mutations in the *TET2* gene are more prone to show hypermethylation of the promoter of the gene and lower gene expression as a result of impaired demethylation of the genome. Moreover, the down-regulation detected was peculiar to the genes involved in sequence-specific DNA binding and chromosomal rearrangement; in particular, epithelial-stromal interaction 1 (*EPSTI1*) and UBA-like domain containing 1 (*UBALD1*) genes showed hypermethylation in *TET2* mutated tissues [44].

In addition to recent genome-wide investigations, several previous studies based on the candidate gene approach support the fact that aberrant gene methylation was more frequent in TCs than in TMs, as also discussed in the previous section. For example, it has been observed that the frequency of methylation of secreted protein acidic and cysteine rich (*SPARC*), death-associated protein kinase (*DAPK*), *CDKN2A*, *MGMT*, and hyperpigmentation, progressive, 1 (*HPP1*) genes increased according to the histological type of thymomas with the highest peak in carcinomas, particularly for what concerns *MGMT* methylation, similarly to what has been reported before [95,97]. *MGMT* methylation was further investigated in an independent study showing a higher frequency in TCs than in TMs and advanced Masaoka stages TETs, with loss of expression of *MGMT* protein. The loss of expression of all those tumor suppressor genes can be reconducted to the global hypomethylation that has been detected in advanced-stage (B2/B3/C) TETs compared with low-stage patients, which is independent of clinical features such as gender and MG manifestations [98].

Genes belonging to the epigenetic machinery are commonly more frequently mutated in TCs than in TMs, affecting epigenetic homeostasis [127]. For instance, DNMTs were found to be significantly upregulated in WHO type B2/B3/C and Masaoka stages, while no difference in the methylation levels of DNA methyltransferase genes was detected between TMs and adjacent healthy tissues [98]. Moreover, the expression of DNMT3b negatively correlates with the reductions of h5MC content, suggesting its role in the development of the worst forms of TETs [98].

In summary, TCs show a distinct methylation landscape with respect to TMs and a worse clinical outcome. Studies in TCs suggest that DNA methylation signatures could represent useful prognostic biomarkers in TET patients.

## 5. Non-Coding RNAs in Thymomas

During the last decades, the different classes of non-coding RNAs—including microRNAs (miRNA)s, small/long non-coding RNAs (snc/lncRNAs), or circular RNAs (cirRNAs)—have been implicated in the onset and development of various malignancies, and for these reasons, ncRNAs have been extensively investigated as potential peripheral biomarkers of those diseases [128,129]. Their importance in thymocyte biology supports the evidence that their dysregulation could be deleterious in certain conditions and promote TET progression [130]. Table 2 and Table 3 show, respectively, a summary of the studies investigating miRNAs and lncRNAs in TETs.

### 5.1. Thymomas

RNA sequencing conducted on tumor biopsies revealed two miRNA clusters (MC) located respectively in chromosome 19q13.42 (C19MC) and chromosome 14q32 (C14MC) that are strongly discriminatory for TET histological subtypes. In particular, the C19MC expression is detectable in A/AB thymomas while it results in completely silenced in B-type thymomas and in TCs [135,139]. Intriguingly, C19MC is an imprinted-active cluster in the placenta that is silenced in adult tissues with the exception of both medullary and cortical thymic epithelial cells [153,154]. It has been suggested that this cluster might be regulated by promoter methylation and its expression results in the activation of PI3K/AKT pathways [139].

The C14MC cluster is another miRNA cluster located within an imprinted chromosomal region, often dysregulated in human cancers [155,156,157,158]. This cluster of miRNAs is downregulated in TCs with respect to A- and B-type TMs, albeit the expression of some miRNAs of the cluster has been detected in TCs [139].

Moreover, different works tried to compare the differences in miRNA expression profiles that occur among TM histotypes, normal tissues, and clinical features of the patients (Table 2). For example, the microarray analysis of two large retrospective cohorts of TETs identified 87 miRNAs as differentially expressed between tumor and healthy tissues [132]. Among the most significant miRNAs identified, hsa-mir-141-3p, hsa-mir-205-5p, hsa-mir-205-3p, hsa-mir-34c-5p, hsa-mir-455-5p, hsa-mir-148a-3p, hsa-mir-34b-5p, hsa-mir-34a-5p, and hsa-mir-21-5p were the 9 miRNAs found upregulated and hsa-mir-451a, hsa-mir-145-5p, hsa-mir-486-5p, hsa-mir-630, and hsa-mir-1207-5p were the 5 miRNAs found significantly downregulated in TMs with respect to healthy thymic tissues. Moreover, the pathways analysis revealed that all these miRNAs were linked to specific cancer-related pathways, including cell adhesion and growth [132]. Moreover, hsa-mir-489 has been found discriminatory for the histological TM subtypes (A/AB/B1 vs. B2/B3). A subsequent investigation was aimed to evaluate the expression of 6 miRNAs—that were previously identified as differentially expressed between tumor and healthy tissues in the Ganci study—on plasma and serum of TET patients. Hsa-mir-21-5p and 148a-3p were found significantly upregulated also in the plasma of the TET patients compared with healthy donors. The importance of these circulating onco-miRNAs as peripheral biomarkers was corroborated by the evidence that their expression resulted as decreased during the following 3 years from the surgery [133]. More recently, based on expression data from the TCGA database, it was observed that hsa-mir-130b-5p, hsa-mir-1307-3p, and hsa-mir-425-5p, were among prognostic factors for relapse-free survival and OS in TMs [142].

In addition, some investigators focused on specific miRNAs dysregulated in TAMG patients [100,105,134,136]. A very recent systematic review analyzed the peculiar dysregulation of miRNAs expression in the blood of MG patients [159]. The meta-analysis revealed a large heterogeneity among different studies, with a total of 226 dysregulated miRNAs in the various studies, but only a few of them recurrently dysregulated in at least three different works [159]. A few studies included TAMG patients in order to assess TAMG-specific miRNA signatures. An increased expression of hsa-mir-19b-5p was observed in thymomas from TAMG patients, and cell culture investigations revealed that this miRNA targets the thymic stromal lymphopoietin (TSLP), an interleukin-7-like cytokine involved in the development and maturation of T cells [134]. Others investigated hsa-mir-20b expression levels and found that it was downregulated in both thymomas and serum from patients with TAMG compared, respectively, to adjacent nontumor tissues and healthy blood samples [136]. The development of MG in TM patients is influenced by the ability of the tumor in promoting the proliferation, maturation, and export of T cells, and the overexpression of hsa-mir-20b inhibits T cell proliferation and activation [160]. Another study observed 137 dysregulated miRNAs in the normal tissue adjacent to the thymoma from TAMG patients with respect to normal thymic tissues from healthy controls. Among them, a dysregulated hsa-mir-125a-5p expression was confirmed in a larger cohort of patients and found to inversely correlate with the expression levels of *Foxp3*, a master regulator of T regulatory cell development and function [137].

Concerning lncRNAs, a recent study observed that AFAP1-AS1, LINC00324, ADAMTS9-AS1, VLDR-AS1, LINC00968, and NEAT1 are deregulated in 25 TMs compared with 25 normal tissues. Of particular interest is the altered regulation of AFAP1-AS1 and LINC00324, which was found to be closely related to patient survival [144]. Moreover, a lncRNA classifier was recently suggested for predicting recurrence-free survival in TET patients. The study included 114 thymoma cases from the TCGA database, of which 19 developed recurrence during a median follow-up of approximately 3 years [143]. In particular, HSD52, LINC0098, ADAMTS9-AS1, and LNC01697 were proposed as viable prognostic factors for low- and high-risk recurrence outcomes, independently from Masaoka stages (I-II vs. III-IV) or TET histotype (TMs vs. TC) [143].

A recent study compared thymomas from patients with and without MG, revealing 4360 lncRNAs and 2545 mRNAs as differentially expressed between TAMG and non-TAMG thymomas. Albeit the study suggested several specific dysregulated pathways in TAMG versus non-TAMG tissues, it was limited by the analysis of only eight thymomas in total [145]. Another recent elaboration of TCGA data concerning the TAMG cohort revealed 56 and 84 lncRNAs, respectively, over and downregulated compared with non-MG thymomas. Interestingly, the immune-related lncRNAs AP000787.1, AC004943.1, WT1-AS, and FOXG1-AS1 expression are regulated by the methylation of the promoter regions [150]. Moreover, the competing endogenous RNA (ceRNA) network LINC00452/miR-204/CHST4 was recently proposed as a crucial axis, which regulates thymic regulatory T cells (Tregs) and the development of TAMG [151].

Moreover, 73 circRNAs resulted in differentially expressed in TMs with respect to adjacent normal tissues. In particular, hsa_circ_0001173, hsa_circ_0007291, hsa_circ_0003550, and hsa_circ_0001947 resulted in significantly upregulated in thymomas and the expression of the parental gene of hsa_circ_0007291 and hsa_circ_0001947 was associated with progression-free survival [146].

In summary, several ncRNAs have shown a peculiar expression in TMs compared to normal tissues. Clustered miRNA expression is specific for TET histologic subtypes. Similarly, the expression levels of many non-clustered ncRNAs can allow distinguishing TAMG from other TMs and could represent useful prognostic biomarkers for relapse-free survival and OS in TM patients.

### 5.2. Thymic Carcinomas

The dysregulation of specific ncRNAs is accountable for the pathophysiology of thymic carcinoma (Table 2 and Table 3). For instance, among the clustered miRNAs, the C19MC locus results completely silenced in TCs, while the C14MC is significantly downregulated albeit several regions remain activated to some degree [139].

High-throughput sequencing technologies allow the identification of a plethora of non-clustered ncRNAs. For instance, a large study of 123 TET patients, 9% of which were affected by TC, identified 19 miRNAs that were upregulated and 72 that were downregulated in TCs. The different expression profiles of TCs allowed us to differentiate them from other histological types with a very low misclassification rate (from 1.6 to 3.3%) [140].

Enkner and colleagues (2017) [139] also reported the TC-specific downregulation of has-mir-34hashsa-mir-34c, hsa-mir-130a, and hsa-mir-195, and the overexpression of hsa-mir-21, hsa-mir-9-3 and hsa-mir-375 in 34 paraffin-embedded TCs compared to 18 type A TMs. These are non-clustered miRNAs with a putative tumor suppressor or oncogenic activity [139].

Moreover, a microarray analysis conducted on formalin-fixed paraffin-embedded (FFPE) biopsies highlighted the different miRNAs expression among different TM histological types compared with carcinomas. In particular, 10 miRNAs were found differentially expressed in C vs. B2/B3 and 4 in C vs. A/AB/B1 histotypes [132]. Of note, the thymic carcinoma-associated miRNAs hsa-mir-142-5p, hsa-mir-363-3p, and hsa-mir-16-2-3p, identified as downregulated in TC, target baculoviral IAP repeat containing 3 (*BIRC3*), small inducible cytokine subfamily A, member 20 (*SCYA20*), and *MYC* mRNAs; these mRNAs are linked to anti-apoptotic signatures that were found co-upregulated in thymic carcinoma cells [131]. Moreover, specific TC immune-related miRNAs (hsa-mir-130b-5p, hsa-mir-1307-3p, and hsa-mir-425-5p, which were all markedly increased in TC compared with A-AB or B1-B3 types) could modulate immune microenvironment and are useful as biomarkers for poor prognosis [142]. An independent study conducted by Bellissimo et al., 2017 [138] analyzed the involvement of miR-145-5p in TETs behavior in an FFPE cohort, which was composed of five TCs and fourteen TMs. According to their results, the expression levels of 69 genes were inversely correlated to those of hsa-mir-145-5p, which could participate in the maintenance of TET by affecting different signaling pathways, including the signaling networks related to immune-related functions (e.g., cytokine–cytokine receptor pathways were downregulated in tumoral vs. normal specimens). Moreover, the inhibition of HDAC activity by means of valproic acid (VPA) upregulates hsa-mir-145-5p expression in the in vitro TC model, and the induction of G1 and migration arrest and cell death 96 h after VPA treatment [138]. These results suggest the epigenetic regulation of hsa-mir-145-5p and its contribution to the tumor phenotype, and also suggest a valuable target for a therapeutic approach to improve the treatment response of TETs [138]. Interestingly, the lncRNA LINC00174 sponges and inhibits the activity of hsa-mir-145-5p, and the in vitro silencing of LINC00174 reduced cell proliferation, migration, and lipid metabolism in a TC cell model (TC1889) [147]. It was also observed that the lncRNA metastasis-associated-lung-adenocarcinoma-transcript-1 (MALAT1) sponges miR-145-5p in TETs [148]. Furthermore, the connection between MALAT1 and the methyltransferase METTL3 that catalyzes the N6-methyladenosine (m6A) modification was described in TC1889 [149]. m6A modification of MALAT1 is critical for the metastatic ability of cancer cells, reshaping the nuclear speckles. The downregulation of METTL3 leads to increased localization of MALAT1 in nuclear speckles and decreased m6A modification of MALAT1, which probably affects its functional activity [161].

An elegant experiment including in vitro/in vivo models, as well as TGCA data, has extensively characterized the miR-525-5p, which promotes apoptosis and inhibits invasion by directly targeting the 3′ UTR mRNA of the target gene *HSPA9*. The levels of miR-525-5p and HSPA9 were inversely correlated in TET tissues compared with normal ones, leading to a poor prognosis and a lower 5-year survival rate. Interestingly, the authors proposed and validated the role of lncRNA LOXL1-AS1 in acting as a sponge for miR-525-5p, promoting *HSPA9* expression and tumor-aggressive phenotype [152].

All these works revealed several ncRNAs that were specifically dysregulated in TCs with respect to other TETs. These ncRNAs have a putative tumor suppressor or oncogenic activity, which could modulate the tumor microenvironment and could represent useful prognostic biomarkers.

## 6. Histone Tail Modifications in TETs

With respect to DNA methylation and ncRNA investigations, which have been extensively performed in TET samples, evidence of the involvement of histone PTMs in TETs is often indirect and suggested by several studies that show different expression of proteins involved in PTMs of histone tails in TETs and other cancers. Similarly, comparisons between TMs and TCs are not available, so the present section is not divided into TMs and TCs.

Histone tail modifications have been implicated in several processes of thymus physiology [162,163]. For instance, *AIRE* is important for a successful negative selection avoiding the release of autoreactive T cells in the periphery by orchestrating the expression of several genes. This regulation occurs by means of chromatin rearrangement. *AIRE* interacts through its PHD finger domain (plant homodomain) with the N terminal ends of H3, sensing the modification occurred and thus mediating transcriptional activation by increasing H3K4me3 and AcH3 levels of target gene promoters [164]. Interestingly, a positive correlation between *AIRE* expression and H3K4me3 was observed in the *AIRE* promoter in both human and mouse thymic epithelial cells, suggesting that *AIRE* expression itself is regulated by histone tail modifications [123].

Moreover, the importance of the tumor microenvironment (TME) and the onset and development of cancer was recently investigated in a study comprising 9075 individuals affected by different cancer types, including thymoma, from the TCGA Pan dataset [165]. The results revealed 1811 mutated sites of PTMs in matrisome genes (genes coding for proteins of the extracellular matrix (ECM) or for ECM-associated proteins), including ubiquitination, sumoylation, methylation, and acetylation, among many others. The perturbation of the regulation of these genes leads to a systemic disruption of the physiological crosstalk between TME and extracellular matrix (ECM) with a subsequent deregulation of potential tumor driver genes. Additionally, an independent study on a large TCGA cohort, including different tumors, investigated the role of the lysine demethylase 4B (*KDM4B*) gene overexpression in hypoxic TME in promoting angiogenesis and metastases formation. In this context, no differences were found in *KDM4B* expression in thymoma tissues compared with healthy tissue among the cancer stages [166]. An inverse correlation between mineral dust-induced (*MDIG*) gene levels and OS of TM patients was detected, as well as a positive correlation with immunostimulatory genes [167,168]. MDIG proteins contain a JmjC domain belonging to the JmjC family of histone demethylases that are able to regulate the trimethylation of H3K9, promoting cell growth and cell cycle progression [167]. Furthermore, the histone deacetylase HDAC6 and the zinc finger E-box-binding homeobox 1 (ZEB1) proteins were shown as differentially expressed in several cancers and are involved in tumor immune infiltration and TME [167,169]. The pan-cancer analysis based on TCGA, genotype-tissue expression (GTEx), and the Cancer Cell Line Encyclopedia (CCLE) datasets revealed that the low expression of *HDAC6* is a potential biomarker of worse prognosis in several cancers, including thymoma [167].

Moreover, a biomarker for a good prognosis of TM patients is the upregulation of *RP11-424C20.2* pseudogene and *URPH1* [170]. *URPH1* is correlated with T-cell-related processes; thus, the authors proposed its control in the interaction of immune and cancer cells. *URPH1* is widely involved in the coordination of several chromatin modifiers and DNA methylation proteins, by which it promotes the silence of several genes, including tumor suppressor genes [171].

Of note, most of the works reported have been conducted by exploring data from datasets such as TCGA or GTEx, and none of them reported the clinical characteristics of the patients.

Despite that evidence of the involvement of histone PTMs in TETs is often indirect, several histone PTMs have been detected in hematopoietic malignancies and solid tumors. This has led to the development of various epigenetic drugs, including histone acetyltransferase and deacetylase inhibitors, histone methyltransferase and demethylase inhibitors, and inhibitors of other histone modifier enzymes. Four of these compounds received FDA approval for the treatment of hematopoietic malignancies, one was approved for the treatment of epithelioid sarcoma, and many others are in preclinical or clinical trials for solid tumors [172]. In line with this, it is important to note that there are several clinical trials that are testing the efficacy of drugs that target HDAC activity with promising results, in particular for TMs patients [138,173,174,175].

## 7. Epigenetic Deregulation of Neuroendocrine Thymic Epithelial Tumors

As described in the introductory section, NETT is the rarest form among thymic malignancies, particularly the one with the most aggressive biological behavior [176].

To the best of our knowledge, only one research article has investigated the epigenetic deregulation that characterized NETTs, to date [101]. In particular, *RASSF1A* exhibited the highest promoter methylation levels in NETT samples with respect to TCs, and type B3 TMs. Additionally, a significant decrease in the *RASSF1A* protein production was observed in NETT specimens, similar to what had previously been reported in other cancer types [101]. Thus, the tumor suppressor activity of *RASSF1A* was confirmed in various malignancies. Considering its involvement in cell cycle control, mitotic arrest, and apoptosis, cancers showing high methylation levels of *RASSF1A* promoter and low protein levels have been frequently characterized with a negative prognosis [177,178,179].

Recently, a genome-wide DNA methylation of 96 samples of various neuroendocrine tumors (NET) has demonstrated that the methylation profiles are unique in different primary NET types associated with hereditary syndromes, MEN1 and VHL [180]. Despite NETT not being included in the study cohort, it shares with them the same primary tumor site and the predisposition for multiple endocrine neoplasia type 1 (MEN1). Another recent investigation proposed a set of miRNAs that could be useful to discriminate among the lung neuroendocrine cancer types that are characterized by subtle histological differences [181]. Similarly, miRNAs are emerging as valuable biomarkers in different types of neuroendocrine malignancy, because of their tissue specificity and as prognostic markers [178,182,183].

## 8. Conclusions

The malignancies of the thymus are a very rare form of mediastinal tumors, in particular, the neuroendocrine forms. Thanks to the high throughput technologies and the available data from big datasets (e.g., TCGA, GTEX), the results from clinical research may be more readily available by facilitating an understanding of the mechanisms underlying rare cancer types. Through the use in large part of these resources, it was possible to determine the burden of the contribution of epigenetic regulation in tumor development and their usefulness in clinical practice as peripheral biomarkers. In particular, DNA methylation and ncRNAs are specific epigenetic signatures with the potential to discriminate among the different histological types and could represent promising diagnostic/prognostic biomarkers. In addition, histone PTMs represent promising therapeutic targets in TETs, and nowadays, several clinical trials are testing the efficacy of drugs targeting the activity of HDACs in these malignancies [138,173,174,175].

Epigenetic investigations in TETs, reviewed in this article, clearly show distinct epigenetic profiles in TMs compared with TCs and suggest that TAMG might represent a distinct epigenetic subtype. A previous TETs classification using multi-omic data from the TCGA dataset, including genetic, cytogenetic, epigenetic, and gene expression data, revealed that TETs have a very low mutation burden, with *GTF2I* being the most frequently mutated gene, particularly in A and AB TMs [43]. Similarly, A and AB TMs overexpress the C19MC. Type B thymomas are more commonly associated with MG than type A or AB TMs, and both type B TMs and TCs tend to overexpress proto-oncogenes and downregulate tumor suppressor genes [43]. Moreover, which study showed that MG-positive TMs were enriched in aneuploidies but not associated with particular mutations or epigenetic signatures. TCs showed often loss of chromosome 16q and an increased mutation burden than TMs [43]. Apart from those integrated analyses from the TCGA dataset, large studies combining the genetic and epigenetic landscape of TETs are missing. Previous investigations performed by us in TAMG samples revealed that most of the genes reported to be hypermethylated in other TETs were largely hypomethylated in this cohort, further supporting evidence that TAMG might be a distinct molecular subtype of TETs [104,105]. Moreover, many previous studies support the hypermethylation of tumor suppressor genes in TCs with respect to early-stage TMs [83,98,99]. Distinct epigenetic profiles among TMs without MG, TAMG, and TCs are also emerging from the analysis of ncRNA expression [131,132,139,145]. Available epigenetic data on NETTs are still scarce [101].

Chemotherapy can be used as a neoadjuvant approach to bring back to resectability locally advanced stage III TMs and represents the first-line treatment for advanced and metastatic TETs, with objective response rates ranging from 50 to 90%. The molecular characterization of TETs, combining information on the genetic and epigenetic landscape, could help to identify biomarkers of response to standard therapeutic compounds. Indeed, promising methylation biomarkers of response to platinum-based chemotherapy have been recently identified in other cancers [184,185].

Immunotherapy can lead to serious autoimmune complications in TET patients, and is therefore considered only in some cases of TC [52]. TETs are programmed death-ligand 1 (PD-L1) expressing tumors [52]. Preclinical models of melanoma and lung adenocarcinoma showed that the expression of PD-L1 and T cell chemokines can be upregulated by HDAC inhibitors to enhance the sensitivity of the immune response to anti-PD-1/PD-L1 therapy and improve clinical outcomes [186]. Moreover, several TETs dysregulated ncRNAs, such as has-miR-130b-5p, has-miR-1307-3p, and has-miR-425-5p, are involved in immune-related pathways [95]. Therefore, we cannot exclude that the information gained from the epigenetic investigation of TETs could lead to improved targeted immunotherapeutic approaches for TETs.

In summary, there is a need for larger studies combining genetic, epigenetic, and expression data from TETs in order to further characterize the different molecular subtypes and identify, for each of them, the most relevant epigenetic biomarkers of clinical utility.

## Figures and Tables

**Table 1:** Works investigating the methylation status of TETs.

Investigated Genes	Cohort	Tissues	Methods	Findings	Ref.
Cyclin-dependent kinase inhibitor 2A (*CDKN2A*)	36 TMs 3 TCs	Tumor tissue samples	MSP	Hypermethylation of *CDKN2* was observed in 4 TMs and 1 TCs.	[94]
Hyperpigmentation, progressive, 1 (*HPP1*), secreted protein acidic and cysteine rich (*SPARC*), Reprimo (*RPRM*), retinoic acid receptor (*RAR6*), ras association domain family member 1 (*RASSF1A*), cadherin 13 (*CDH13*), pl6 (*CDKN2A*), secretoglobin family 3A member 1 (*SCGB3A1*), cadherin 1 (*CDH1*), gremlin 1 (*GRM1*), *RAD*	6 TMs 5TCs	Tumor and adjacent NT samples	MSP	The overall methylation pattern was higher in TC.	[95]
F-box and WD repeat domain containing 7 (*FBXW7*)	13 TMs	Tumor tissue samples	Bisulphite sequencing	*FBXW7* b-form promoter was hypermethylated in types B1 TMs or higher histotypes.	[96]
Death-associated protein kinase 1 (*DAPK*), p16 (*CDKN2A*), O-6-methylguanine-DNA methyltransferase (*MGMT*), hyperpigmentation, progressive, 1 (*HPP1*)	26 TMs 6 TCs	Tumor tissue samples	MSP	The investigated genes were more methylated in TCs than in TMs (60% vs. 20%). *MGMT* methylation was detected in 23% of TMs and in 83% of TCs.	[97]
Global methylation (5 mC content) 9 candidate TSGs	65 TETs	40 PEFF samples and 25 fresh frozen tissues	ELISA (for global methylation) Nested MSP (for gene-specific methylation)	Global DNA methylation levels were reduced, and DNA methyltransferase expression was increased in advanced-stage TETs. E-cadherin (*E-cad*), retinoic acid receptor beta (*RARβ*), human mutL homolog 1 (*hMLH1*), *RASSF1A*, adenomatous polyposis coli promoter 1A (*APC1A*), and fragile histidine triad diadenosine triphosphatase (*FHIT*) genes were hypermethylated in B2/B3/C TMs relative to A/AB/B1 TMs.	[98]
*MGMT*	66 TETs	Tumor tissue samples	MSP	*MGMT* methylation was more frequent in TCs than in TMs, also correlated with loss of protein expression.	[99]
Methylenetetrahydrofolate reductase (*MTHFR*), DNA methyltransferases (*DNMT1*, *DNMT3A*, *DNMT3B*)	69 TAMGs	Tumor tissue and 44 paired NT samples. Blood samples	MS-HRM	*MTHFR* and *DNMT3A* promoters were more methylated in tumor tissue with respect to blood. *MTHFR* promoter was more methylated in tumor tissue respect to healthy adjacent thymic epithelial cells. *DNMT1* and *DNMT3B* promoters were hypomethylated in all tissues.	[100]
*RASSF1A*	8 TMs 6 TCs 3 NETs	Tumor tissue and adjacent NT samples	Bisulphite pyrosequencing	*RASSF1A* was hypermethylated in NETs, but not in TCS or TMs.	[101]
GWAS	124 TETs	TCGA dataset	Illumina Infinium HumanMethylation450K	5155 CpG sites hypomethylated and 6967 CpG sites hypermethylated in TMs and TCs. High methylation in kinase suppressor of Ras 1 (*KSR1*), E74-like ETS transcription factor 3 (*ELF3*), interleukin 1 receptor antagonist (*IL1RN*) correlates with a good prognosis; low methylation in recombination activating 1 (*RAG1*) correlates with longer OS.	[102]
GWAS	6 TETs	Tumor tissue and adjacent NT samples	Infinium MethylationEPIC BeadChip microarray (850 K)	119 hypermethylated and 18,999 hypomethylated DMCs in tumor vs. normal tissue. 7 hypermethylated and 29 hypomethylated genes. Fasciculation and elongation protein zeta 2 (*FEZ2*), protein tyrosine phosphatase receptor type E (*PTPRE*), ATPase sarcoplasmic/endoplasmic reticulum Ca2+ transporting 2 (*ATP2A2*), Cbl proto-oncogene B (*CBlB*), chromosome 5 open reading frame 46 (*C5orf45*), carboxypeptidase E (*CPE*), follistatin-like 1 (*FSTl1*), zinc finger protein 396 (*ZNF396*), fraser extracellular matrix complex subunit 1 (*FRAS1*), neuron navigator 2 (*NAV2*) and lebercilin *LCA5* (*lCA5*) may be potential biomarkers for the diagnosis of type A and type B thymomas.	[103]
*MLH1*, *MGMT*, *CDKN2A*, *RASSF1A*	69 TAMGs	Tumor tissue and 44 paired NT samples. Blood samples	MS-HRM	*MLH1*, *MGMT*, *CDKN2A*, and *RASSF1A* genes promoter methylation were not increased in TAMGs with respect to healthy tissues.	[104]
Growth hormone secretagogue receptor (*GHSR*)	65 TAMG	Tumor tissue and 43 paired NT samples. Blood samples	MS-HRM	*GHSR* hypermethylation was observed in 18 TMs compared to the healthy thymic tissues and demethylated in the remaining 47 TMs.	[105]
GWAS	46 TETs	Tumor tissue and 20 paired adjacent NT samples.	Illumina HumanMethylation450 K BeadChip, pyrosequencing	92 CGI were hypermethylated in the TCs with respect to B3 TMs. 5 CpG sites in the *GHSR* gene were more methylated in TMs and TCs than in the thymus. G protein subunit gamma 4 (*GNG4*), spalt-like transcription factor *3* (*SALL3*), and homeobox D9 (*HOXD9*) DNA methylation rates were significantly higher in TC than in the thymus.	[106]
KIT proto-oncogene ligand (*KITLG*)	121 TMs + 37 TMs *	TCGA/GEO datasets	Methylation data from TCGA/GEO datasets	*KITLG* overexpression is associated with 28 hypermethylated and 163 hypomethylated regions.	[107]
*MTHFR*	123 TETs	TCGA dataset	Methylation data from TCGA dataset	Negative correlation between the transcriptional and methylation levels of *MTHFR.*	[108]
Glutamate decarboxylase 1 (*GAD1*)	73 TMs 17 TCs	47 tumor tissues and 21 paired adjacent NT samples	Pyrosequencing	*GAD1* hypermethylation is higher in TCs than TMs.	[109]
GWAS	TCGA cohort	TCGA dataset	Methylation data from TCGA dataset	943 DMCs between TMs and controls; 14 genes, including *lipase E* (*LIPE*) resulted epigenetically regulated.	[110]
Mitogen-activated protein kinase 4 (*MAPK4*)	124 TETs from TCGA 95 TET *	Tumor tissues	TCGA dataset/pyrosequencing	*MAPK4* methylation is a prognostic factor for RFS.	[111]
MARVEL domain containing 1 (*MARVELD1*)	10,534 patients representing 33 cancer types	TCGA dataset	Methylation data from TCGA dataset	Hypermethylation in the *MARVELD1* promoter locus synergistically affected its expression in pan-cancer.	[112]

Thymic epithelial tumor (TET); thymoma (TM); thymic carcinoma (TC); normal tissue (NT); 5-methyl cytosine (5 mC); paraffin-embedded formalin-fixed (PEFF); methylation-specific PCR (MSP); methylation sensitive high-resolution melting (MS-HRM), tumor suppressors gene (TSG); The Cancer Genome Atlas (TCGA); Gene Expression Omnibus (GEO) datasets; genome-wide association study (GWAS); replication cohort (*).

**Table 2:** Works investigating microRNAs in TETs.

Investigated ncRNA	Cohort	Tissues	Methods	Findings	Ref.
MiRNome	51 TMs 8 TCs 7 NT + 36 TMs * 11 TCs	Tumor and NT sample	Data from the TCGA dataset	Hsa-mir-142-5p, hsa-mir-363-3p, and hsa-mir-16-2-3p were dysregulated in TC and linked to antiapoptotic pathways.	[131]
MiRNome	54 TETs, 12 NT	FFPE tissues	Microarray analysis	87 miRNAs differently expressed between TETs and normal samples. Hsa-mir-7-5p, hsa-mir-489, hsa-mir-1208, hsa-mir-1323, hsa-mir-519e-5p, hsa-mir-516b-5p, hsa-mir-921, hsa-mir-509-5p, hsa-mir-138-2-3p, and hsa-mir-342-5p were dysregulated in TCs vs. B2/B3 TMs. Hsa-mir-650, hsa-mir-181c-5p, hsa-mir-363-3p, hsa-mir-181a-3p were dysregulated in TCs vs. A/AB/B1 TMs.	[132]
MiRNome	5 TETs	Blood samples	RT-qPCR	Hsa-mir-21-5p and 148a-3p were found significantly upregulated in plasma of the TET patients.	[133]
hsa-mir-19b	52 TAMG 12 TMs 11 NT	Tumor tissues and healthy thymus	RT-qPCR	Hsa-mir-19b-5p was increased in TMs from TAMG patients.	[134]
C19MC C14MC	13 TET 3 NT	Tumor tissue and NT sample	RNA-seq	C19MC is a genomic hallmark of type A and AB TMs.	[135]
hsa-mir-20b	30 TAMG	Tumor tissue samples and serum	RT-qPCR	Hsa-mir-20b expression level is downregulated in both TMs and serum from patients with TAMG.	[136]
hsa-mir-125a-5p	13 TAMG	Tumor tissues, adjacent NT and normal thymus samples	Microarray analysis	137 miRNAs in NT adjacent to the TM from TAMG patients that were significantly dysregulated compared with normal thymus in controls. Hsa-mir-125a-5p expression negatively correlates with foxp3 expression in NT with respect to the thymoma from TAMG.	[137]
hsa-mir-145-5p	14 TMs 5 TCs	FFPE tissues	Microarray analysis	Hsa-mir-145-5p contributes to the tumor phenotype.	[138]
C19MC	37 TMs 35 TCs 6 NT	FFPE tissues	RNA-seq	C14MC and C19MC miRNA clusters are highly expressed in type A thymomas but silenced in TCs. Hsa-mir-21, hsa-mir-9-3 and hsa-mir-375 and hsa-mir-34b, hsa-mir-34c, hsa-mir-130a, and hsa-mir-195 were respectively upregulated and downregulated in TCs.	[139]
MiRNome	123 TETs (9% TCs)	Tumor tissues sample	Data from the TCGA dataset	9 miRNAs upregulated and 72 miRNAs downregulated in TCs. Let-7a-1, let-7a-2, let-7a-3, hsa-mir-140, hsa-mir-324, hsa-mir-639, and hsa-mir-3613 dysregulation was associated with DFS or OS in TCs.	[140]
hsa-mir-27-a	15 TETs	Tumor and normal tissues sample	RT-qPCR	Hsa-mir-27-a is upregulated in TC tissues. Curcumin treatment decrease hsa-mir-27-a levels in TC1889 cells.	[141]
MiRNome	119 TETs + 99 TETs *	FFPE tissues	RNA seq from TCGA dataset	Hsa-mir-130b-5p, hsa-mir-1307-3p, and hsa-mir-425-5p, were among prognostic factors for RLFS and OS in TCs.	[142]

Thymic epithelial tumor (TET); thymoma (TM); normal tissue (NT); thymoma associated with myasthenia gravis (TAMG); quantitative real-time PCR (RT-qPCR); RNA sequencing (RNA-seq); formalin-fixed paraffin embedded (FFPE); competing endogenous RNA (ceRNA) network; progression-free survival (PFS); Relapse-free survival (RLFS); disease-free survival (DFS); overall survival (OS); validation cohort (*); quantitative real-time PCR (RT-qPCR).

**Table 3:** Works investigating the long non-coding RNAs in TETs.

Investigated lncRNA	Cohort	Tissues	Methods	Findings	Ref.
Whole LncRNA analysis	114 TMs from TCGA	Tumor tissue samples	RNA-seq	HSD52, LINC0098, ADAMTS9-AS1, and LNC01697 were proposed as a viable prognostic factor for low- and high-risk recurrence outcomes.	[143]
Whole LncRNA analysis	25 TMs 25 NT	Tumor tissue samples	RNA-seq	AFAP1-AS1, LINC00324, ADAMTS9-AS1, VLDR-AS1, LINC00968, and NEAT1 are deregulated in TMs compared to NT.	[144]
Whole LncRNA analysis	5 TMs 3 TAMG	Tumor tissue samples	Microarray analysis	4360 lncRNAs and 2545 mRNAs were differentially expressed between TAMG and non-TAMG thymomas.	[145]
Whole LncRNA analysis	22 TETs	Tumor tissue samples and NT tissues	RNA-seq	The circulating RNA hsa_circ_0001173, hsa_circ_000729.1, hsa_circ_0003550, and hsa_circ_0001947 are upregulated in TMs and associated with PFS.	[146]
LINC00174	119 TETs + 6 TETs * 3 NT	Tumor and NT samples	Microarray analysis/ Data from the TCGA dataset	LINC00174 is upregulated in TETs and sponges has-mir-145-5p.	[147]
MALAT1	IU-TAB-1 cell line	Cell models	RT-qPCR	MALAT1 silencing attenuated cell proliferation and apoptosis via the miR-145-5p/HMGA2 pathway in TC cell models.	[148]
MALAT1	TC1889 cell line	Cell models	RT-qPCR	c-MYC protein is enabled by MALAT1, which is delocalized by METTL3, acting as a tumor promoter in TCs.	[149]
Whole LncRNA analysis	29 TAMG 58 TETs	Tumor tissues	TCGA dataset	TAMG showed specific up and downregulation of 56 and 84 lncRNAs, respectively. The lncRNA AP000787.1, AC004943.1, WT1-AS, FOXG1-AS1 are shown to be regulated by methylation of their promoter region.	[150]
RNA expression analysis	27 TAMG 67 TMs	Tumor tissues	TCGA dataset	The LINC00425/hsa-mir-204/CHST4 axis is potentially involved in the progression of TAMG.	[151]
LOXL1-AS1	42 TMs 28 TCs	Tumor samples and Thy0517/Ty-82 cell models	RT-qPCR/Data from the TCGA dataset	LOXL1-AS1 sponges hsa-mir--525-5p-HSPA9 target. Downregulation of hsa-mir-525-5p and high levels of LOXL1-AS1 and HSPA9 were associated with poor prognosis in TCs.	[152]

Thymic epithelial tumor (TET); thymoma (TM); normal tissue (NT); thymoma associated with myasthenia gravis (TAMG); quantitative real-time PCR (RT-qPCR); RNA sequencing (RNA-seq); formalin-fixed paraffin embedded (FFPE); competing endogenous RNA (ceRNA) network; progression-free survival (PFS); relapse-free survival (RLFS); disease-free survival (DFS); overall survival (OS); validation cohort (*); quantitative real-time PCR (RT-qPCR).
